# Clinical assessment of bipolar depression: validity, factor structure and psychometric properties of the Korean version of the Bipolar Depression Rating Scale (BDRS)

**DOI:** 10.1186/s12888-016-0958-7

**Published:** 2016-07-15

**Authors:** Young-Eun Jung, Moon-Doo Kim, Won-Myong Bahk, Young Sup Woo, Jonghun Lee, Sae-Heon Jang, Seunghee Won, Kyung Joon Min, Sangkeun Chung, Young-Joon Kwon, Duk-In Jon, Kwanghun Lee, Bo-Hyun Yoon

**Affiliations:** Department of Psychiatry, School of Medicine, Jeju National University, Jeju, Republic of Korea; Department of Psychiatry, College of Medicine, The Catholic University of Korea, Seoul, Republic of Korea; Department of Psychiatry, Catholic University of Daegu, School of Medicine, Daegu, Republic of Korea; Department of Psychiatry, Bongseng Memorial Hospital, Busan, Republic of Korea; Department of Psychiatry, Kyungpook National University School of Medicine, Daegu, Republic of Korea; Department of Psychiatry, College of Medicine, Chung-Ang University, Seoul, Republic of Korea; Department of Psychiatry, Chonbuk National University Medical School, Jeonju, Republic of Korea; Department of Psychiatry, Chunan Soonchunhyang Hospital, Soonchunhyang University, Chunan, Republic of Korea; Department of Psychiatry, Sacred Heart Hospital, College of Medicine, Hallym University, Anyang, Republic of Korea; Department of Psychiatry, College of Medicine, Dongguk University, Gyeongju, Republic of Korea; Department of Psychiatry, Naju National Hospital, Naju, Republic of Korea

**Keywords:** Bipolar disorder, BDRS, Bipolar depression, Korean, Reliability, Validity

## Abstract

**Background:**

The Bipolar Depression Rating Scale (BDRS) is a scale for assessment of the clinical characteristics of bipolar depression. The primary aims of this study were to describe the development of the Korean version of the BDRS (K-BDRS) and to establish more firmly its psychometric properties in terms of reliability and validity.

**Methods:**

The study included 141 patients (62 male and 79 female) who had been diagnosed with bipolar disorder, were currently experiencing symptoms of depression, and were interviewed using the K-BDRS. Other measures included the Montgomery and Asberg Depression Scale (MADRS), the 17-item Hamilton Depression Scale (HAMD), and the Young Mania Rating Scale (YMRS). Additionally, the internal consistency, concurrent validity, inter-rater reliability, and test-retest reliability of the K-BDRS were evaluated.

**Results:**

The Cronbach’s α-coefficient for the K-BDRS was 0.866, the K-BDRS exhibited strong correlations with the HAMD (*r* = 0.788) and MADRS (*r* = 0.877), and the mixed symptoms score of the K-BDRS was significantly correlated with the YMRS (*r* = 0.611). An exploratory factor analysis revealed three factors that corresponded to psychological depressive symptoms, somatic depressive symptoms, and mixed symptoms.

**Conclusions:**

The present findings suggest that the K-BDRS has good psychometric properties and is a valid and reliable tool for assessing depressive symptoms in patients with bipolar disorder.

## Background

A majority of patients with bipolar disorder experience depressive symptoms throughout approximately half of their lives [1, 2], and as a result, are often misdiagnosed with unipolar depression (depressive disorder) [3]. The failure to correctly diagnose these patients may lead to inappropriate treatment that can worsen the course of the disease [4] and, thus, a diagnostic approach for the recognition of bipolar disorder is essential. Nevertheless, it is difficult to distinguish bipolar depression from unipolar depression in real clinical practice settings. Active inquiry regarding past mania or hypomania is essential to diagnosis, but there are often no distinct prior episodes of mania to be described by the patient.

Several studies have suggested that there is a broad range of symptomatic differences between patients with unipolar depression and those with bipolar depression. Melancholic symptoms (worthlessness, marked anhedonia, and pathological guilt) and atypical depressive features (hypersomnia, hyperphagia, mood reactivity, and leaden paralysis) are more common in bipolar depression than in unipolar depression [5–9]. It has also been found that mixed features such as irritability, mood lability, distractibility, and racing thoughts are more common in patients with bipolar depression [10, 11]. Yet, the widely used scales in clinical and research practice in bipolar depression are the Montgomery and Asberg Depression Scale (MADRS, [12]) and the 17-item Hamilton Depression Scale (HAMD, [13]), which were originally developed for use in unipolar depression. They do not capture many core symptoms of bipolar depression, including atypical and mixed features.

The Bipolar Depression Rating Scale (BDRS) is a semi-structured, observer-rated scale for clinical assessment of bipolar depression. It was developed by Berk et al. [14] based on previous phenomenological research and literature reviews that assessed differences between patients with bipolar depression and those with unipolar depression [7, 8, 15, 16]. The BDRS comprises items that evaluate the clinical features associated with the depressive phase of bipolar disorder, including atypical symptoms and mixed phenomenology [14]. This measure can be used to assess depressive symptomatology as well as to evaluate the effectiveness of therapeutic agents for the treatment of bipolar depression.

The objectives of this study were to develop a Korean version of the BDRS (K-BDRS) while maintaining its basic structure and to evaluate the reliability and validity of this measure in the Korean population.

## Methods

### Patients

This study included 141 patients (62 males and 79 females) who were diagnosed with bipolar disorder according to the criteria of the Diagnostic and Statistical Manual of Mental Disorders, Fourth Edition, Text Revision (DSM-IV-TR) [17] and who were currently experiencing symptoms of depression but did not necessarily fulfill the criteria for a major depressive episode. Bipolar disorder and comorbid psychiatric disorders were diagnosed using the Korean version of the Mini International Neuropsychiatric Interview (MINI) and the DSM-IV-TR [18, 19].

All patients were recruited from 11 university hospitals throughout the territories of the Republic of Korea between 1 September 2013 and 28 February 2015 and were between 18 and 65 years of age. Patients with comorbid psychiatric disorder (*n* = 21), were included if their diagnosis included bipolar disorder as the primary illness. Patients were excluded if there was any evidence of severe cognitive impairment. All patients were receiving standard medications for bipolar disorder, including mood stabilizers (lithium, valproate, carbamazepine, or lamotrigine), atypical antipsychotics (aripiprazole, olanzapine, quetiapine, or risperidone) and antidepressants.

All patients provided informed consent for their participation in this study after the procedure had been fully explained to them and the study protocol was approved by the Institutional Review Boards of the ethical committee of the Jeju National University Hospital at the Jeju University of Korea (JEJUNUH 2013-11-003-001).

### Measurements and procedures

All participants were assessed using the K-BDRS and then interviewed with the Montgomery and Asberg Depression Scale (MADRS, [12]), the 17-item Hamilton Depression Scale (HAMD, [13]), and the Young Mania Rating Scale (YMRS, [20]). These measures were selected to assess the concurrent validity of the K-BDRS. All investigators and raters involved in this study were clinical psychiatrists with more than 10 years of clinical experience in bipolar disorder, and had received formal training in the use of all the rating scales.

The BDRS consists of 20 items that are rated from 0–3 on a Likert-type scale according to a manual that describes the characteristics of each individual item [14]. The BDRS total score ranges from 0–60, such that higher scores reflect more severe depressive symptoms. The original BDRS has good internal consistency (Cronbach’s α = 0.917) as well as strong correlations with other depression rating scales [14].

The original BDRS scale and its manual (freely available at http://www.barwonhealth.org.au/bdrs/) were translated into Korean by two psychiatrists (YEJ and MDK), and then back-translation was performed by a bilingual psychiatrist unaware of the original BDRS. A preliminary translated version was modified until the back-translated version was comparable with the original English version. Three authors of the study (YEJ, MDK, and WMB.) reviewed the results before producing the final version.

The 17-item HAMD is the most widely used instrument for clinical assessment of the severity of depressive symptoms and it is frequently used in studies as an anchor for comparison with novel measures [13]. The Korean version of the HAMD has good psychometric properties [21]. The MADRS is a 10-item rating measure specifically designed to assess treatment-induced changes in depressive symptoms [12]. The Korean version of the MADRS has been confirmed as valid and reliable [22]. The YMRS consists of 11 items and is the most widely used measure for assessment of the severity of manic symptoms [20]. The Korean version of the YMRS has been confirmed as valid and reliable [23].

A subgroup of 20 patients was interviewed by two raters to examine the inter-rater reliability of the K-BDRS. Additionally, to assess test-retest validity, 35 subjects were evaluated by the same investigator who performed the two testing sessions within 5 days.

### Statistical analysis

The internal consistency of the K-BDRS was determined using Cronbach’s α-coefficients. The inter-rater and test-retest reliability values were computed using intraclass correlation coefficients (ICC) [24]. ICC values range from 0 to 1; values of 0.7 and over are considered to indicate ‘substantial agreement’ and values of 0.5–0.7 are considered to indicate ‘moderate agreement’ [25]. The concurrent validity was determined using Pearson’s correlation coefficients to compute the strength and direction of the relationships between scores on the K-BDRS and the other measures. Additionally, a factor analysis was conducted using the unweighted least squares method with oblique factor rotations. A *p* value < 0.05 was considered to indicate statistical significance.

## Results

All relevant demographic and clinical characteristics are presented in Table 1. The internal consistency of the K-BDRS was assessed using Cronbach’s α-coefficient, which was calculated as 0.866. The internal consistency of the depressive subscale of the K-BDRS (Items 1–15) was 0.893 but the mixed subscale (Items 16–20) showed a lower Cronbach’s α-coefficient of 0.756. Table 2 shows the correlations of each item with the total K-BDRS score using a criterion of 0.30 as an acceptable corrected item-total correlation [26]; all but four of the items performed adequately. This lower correlation followed an expected trend for the mixed subscale symptoms such that they correlated less strongly with the total K-BDRS score.

**Table 1:** Demographic and clinical characteristics of the patients

Gender (*n*, %)		
	Male	62 (44.0 %)
	Female	79 (56.0 %)
Age (year, mean ± SD)		38.6 ± 13.7
Years in education (year, mean ± SD)		13.3 ± 3.2
Marital status (*n*, %)		
	Never married	62 (34.3 %)
	Married	63 (34.8 %)
	Divorced/Separated/Widowed	16 (8.9 %)
Diagnosis (*n*, %)		
	Bipolar I disorder	103 (73.0 %)
	Bipolar II disorder	35 (24.8 %)
	Bipolar disorder NOS	3 (2.1 %)
Age of onset (year, mean ± SD)		30.1 ± 13.2
Duration of illness (year, mean ± SD)		8.8 ± 9.0
Medication		
	MS + AAP	97 (68.8 %)
	MS + AAP + AD	21 (14.9 %)
	AAP + AD	11 (7.8 %)
	MS + AD	6 (4.3 %)
	AAP monotherapy	5 (3.5 %)
	MS monotherapy	1 (0.7 %)
Psychiatric comorbidity		
	None	120 (85.1 %)
	Alcohol use disorder	11 (7.8 %)
	Anxiety disorder	8 (5.7 %)
	Eating disorder	2 (1.4 %)
Scales (mean ± SD)		
	K-BDRS score	25.1 ± 9.3
	HAMD score	17.4 ± 8.6
	MADRS score	25.9 ± 10.5
	YMRS score	4.6 ± 5.3

*K-BDRS* Korean version of the Bipolar Depression Rating Scale, *HAMD* Hamilton Depression Scale, *MADRS* Montgomery and Asberg Depression Scale, *YMRS* Young Mania Rating Scale, *SD* standard deviation, *MS* mood stabilizer, *AAP* atypical antipsychotic, *AD* antidepressant

**Table 2:** Internal consistency of the K-BDRS

	Mean value if item deleted	Scale variance if item deleted	Item-total correlation	Cronbach’s Alpha if item deleted
1. Depression	23.05	75.472	.694	.851
2. Sleep disturbance	23.51	80.036	.301	.867
3. Appetite disturbance	23.80	78.981	.353	.865
4. Social impairment	23.41	77.338	.504	.858
5. Activity/energy reduction	23.36	76.059	.630	.854
6. Reduced motivation	23.34	77.189	.569	.856
7. Reduced concentration	23.66	79.047	.497	.859
8. Anxiety	23.76	75.638	.636	.853
9. Anhedonia	23.47	76.870	.615	.854
10. Flattened affect	24.06	79.882	.391	.862
11. Worthlessness	23.48	75.489	.635	.853
12. Helplessness	23.27	72.602	.727	.848
13. Suicidal ideation	23.82	72.795	.649	.851
14. Guilt	23.92	76.922	.571	.856
15. Psychotic symptoms	24.69	80.847	.348	.864
16. Irritability	24.27	83.552	.171	.869
17. Lability	24.04	81.696	.254	.867
18. Increased motor drive	24.78	86.404	−.033	.874
19. Increased speech	24.86	85.893	.023	.871
20. Agitation	24.36	78.720	.458	.860

*K-BDRS* Korean version of the Bipolar Depression Rating Scale

The inter-rater reliability was analyzed in a subsample of patients (*n* = 20) who were interviewed by two different raters. The inter-rater reliability was high for both the total K-BDRS score (ICC = 0.954, 95 % confidence interval [CI] = 0.884–0.982, *p* < 0.001) and the individual items (ICC > 0.70). The test-retest reliability was also analyzed in a subsample of patients (*n* = 35) who had no clinical change between two testing sessions, and was high for both the total K-BDRS score (ICC = 0.950, 95 % CI = 0.901–0.975, *p* < 0.001) and the individual items as well (ICC > 0.70; Table 3).

**Table 3:** Inter-rater and test-retest reliability of the K-BDRS

	Inter-rater reliability		test-retest reliability	
	ICC (95 % CI)	*p*	ICC (95 % CI)	*p*
1. Depression	0.833 (0.579–0.934)	<0.001	0.770 (0.369–0.794)	<0.001
2. Sleep disturbance	0.948 (0.869–0.980)	<0.001	0.969 (0.883–0.938)	<0.001
3. Appetite disturbance	0.945 (0.861–0.978)	<0.001	0.931 (0.863–0.965)	<0.001
4. Social impairment	0.886 (0.712–0.955)	<0.001	0.904 (0.810–0.952)	<0.001
5. Activity/energy reduction	0.807 (0.512–0.923)	<0.001	0.925 (0.852–0.962)	<0.001
6. Reduced motivation	0.760 (0.393–0.905)	0.002	0.834 (0.671–0.916)	<0.001
7. Reduced concentration	0.759 (0.392–0.905)	0.002	0.903 (0.808–0.951)	<0.001
8. Anxiety	0.780 (0.444–0.913)	0.001	0.825 (0.653–0.912)	<0.001
9. Anhedonia	0.831 (0.573–0.933)	<0.001	0.863 (0.728–0.931)	<0.001
10. Flattened affect	0.847 (0.613–0.939)	<0.001	0.916 (0.835–0.958)	<0.001
11. Worthlessness	0.851 (0.623–0.941)	<0.001	0.882 (0.767–0.941)	<0.001
12. Helplessness	0.851 (0.623–0.941)	<0.001	0.805 (0.613–0.901)	<0.001
13. Suicidal ideation	0.915 (0.785–0.966)	<0.001	0.933 (0.868–0.966)	<0.001
14. Guilt	0.819 (0.543–0.928)	<0.001	0.912 (0.825–0.956)	<0.001
15. Psychotic symptoms	0.936 (0.839–0.975)	<0.001	0.959 (0.918–0.979)	<0.001
16. Irritability	0.919 (0.797–0.968)	<0.001	0.868 (0.739–0.934)	<0.001
17. Lability	0.913 (0.781–0.966)	<0.001	0.823 (0.649–0.911)	<0.001
18. Increased motor drive	0.937 (0.840–0.975)	<0.001	0.920 (0.841–0.959)	<0.001
19. Increased speech	0.791 (0.472–0.917)	0.001	0.919 (0.839–0.959)	<0.001
20. Agitation	0.847 (0.614–0.939)	<0.001	0.861 (0.725–0.930)	<0.001

*K-BDRS* Korean version of the Bipolar Depression Rating Scale, *ICC* intraclass correlation coefficient, *CI* confidence interval

The K-BDRS was factor analyzed using the data of all 141 patients to determine the optimal number of factors that described the scale. An unweighted least squares factor analysis was followed by oblique rotations of 2–4 factors. Prior to the rotation, the eigenvalues for the first four factors were 6.67, 3.36, 1.36, and 1.14 with corresponding percentages of variance accounted for of 33.37, 16.79, 6.79, and 5.70 %. The three-factor rotation was determined to provide the most useful description of the data and these three factors were labeled as psychological depressive symptoms (Factor 1), somatic depressive symptoms (Factor 2), and mixed symptoms (Factor 3). Table 4 shows the factor loadings and Table 5 shows the correlations among the total scores of the K-BDRS and the other factor scores.

**Table 4:** Factor loadings of the K-BDRS.

	Factor		
	1	2	3
13. Suicidal ideation	**.794**	−.077	.064
14. Guilt	**.726**	−.097	.015
11. Worthlessness	**.628**	.156	−.053
12. Helplessness	**.603**	.324	−.032
8. Anxiety	**.578**	.128	.213
1. Depression	**.553**	.287	.060
17. Lability	**.500**	−.357	.392
2. Sleep disturbance	**.393**	−.074	−.002
10. Flattened affect	−.289	**.952**	.235
6. Reduced motivation	.250	**.621**	−.194
5. Activity/energy reduction	.296	**.619**	−.140
9. Anhedonia	.272	**.593**	−.026
4. Social impairment	.302	**.410**	−.135
7. Reduced concentration	.266	**.405**	−.014
3. Appetite disturbance	.191	**.213**	.196
18. Increased motor drive	−.192	.049	**.840**
19. Increased speech	−.040	−.086	**.797**
20. Agitation	.196	.287	**.574**
15. Psychotic symptoms	−.015	.412	**.560**
16. Irritability	.380	−.322	**.395**

Extraction method: unweighted least squares

Rotated method: Promax with Kaiser normalization

Loadings in bold represent the highest loading of each item onto one of the three factors

*K-BDRS* Korean version of the Bipolar Depression Rating Scale

Factor 1: psychological depressive symptoms

Factor 2: somatic depressive symptoms

Factor 3: mixed symptoms

**Table 5:** Correlation coefficients among the K-BDRS scores and factor scores (*n* = 141)

	K-BDRS Total	K-BDRS depressive	K-BDRS mixed	Factor 1	Factor 2	Factor 3
K-BDRS total	1					
K-BDRS depressive	0.958**	1				
K-BDRS mixed	0.375**	0.092	1			
Factor 1	0.921**	0.888**	0.328**	1		
Factor 2	0.800**	0.893**	−0.108	0.611**	1	
Factor 3	0.417**	0.167*	0.907**	0.298**	−0.038	1

*K-BDRS* Korean version of the Bipolar Depression Rating Scale, *K-BDRS depressive* K-BDRS scale without mixed symptoms (Items 1–15), *K-BDRS mixed* mixed symptoms of K-BDRS (Items 16–20)

Factor 1: psychological depressive symptoms

Factor 2: somatic depressive symptoms

Factor 3: mixed symptoms

*Correlation is significant at the 0.05 level (*p* < 0.05, two-tailed)

**Correlation is significant at the 0.01 level (*p* < 0.01, two-tailed)

To assess the concurrent validity of the K-BDRS, the total scores and factor scores on the K-BDRS were compared with those on the HAMD, MADRS, and YMRS. The K-BDRS total scores were strongly correlated with the HAMD scores (*r* = 0.788, *p* < 0.001) and the MADRS scores (*r* = 0.877, *p* <0.001), the depression subscale scores of the K-BDRS (Items 1–15) were strongly correlated with the HAMD scores (*r* = 0.888, *p* < 0.001) and the MADRS scores (*r* = 0.759, *p* < 0.001), and the mixed subscale scores (Item 16–20) were strongly correlated with the YMRS scores (*r* = 0.611, *p* < 0.001). The correlations between the K-BDRS factor scores and the total scores on the HAMD, MADRS and YMRS are presented in Table 6.

**Table 6:** Correlation coefficients for the total scores of the K-BDRS, HAMD, MADRS, YMRS, and factor scores

	HAMD	MADRS	YMRS
K-BDRS total	0.788**	0.877**	0.042
K-BDRS depressive	0.888**	0.759**	−0.145
K-BDRS mixed	0.173*	0.283*	0.611**
Factor 1	0.752**	0.841**	0.058
Factor 2	0.606**	0.755**	−0.317**
Factor 3	0.320**	0.206*	0.591**

*K-BDRS* Korean version of the Bipolar Depression Rating Scale, *HAMD* Hamilton Depression Scale, *MADRS* Montgomery and Asberg Depression Scale, *YMRS* Young Mania Rating Scale, *K-BDRS depressive* K-BDRS scale without mixed symptoms (Items 1–15), *K-BDRS mixed* mixed symptoms of K-BDRS (Items 16–20)

Factor 1: psychological depressive symptoms

Factor 2: somatic depressive symptoms

Factor 3: mixed symptoms

*Correlation is significant at the 0.05 level (*p* < 0.05, two-tailed)

**Correlation is significant at the 0.01 level (*p* < 0.01, two-tailed)

## Discussion

The present findings suggest that the K-BDRS has good psychometric properties and may be a reliable and valid tool for measurement of the severity of depressive symptoms in Korean patients with bipolar disorder. Compared with the original BDRS (α = 0.917), the K-BDRS has a somewhat low internal consistency (α = 0.866), but the internal consistency is consistent with other translated versions from Iran (α = 0.81) [27], Turkey (α = 0.786) [28], and Spain (α = 0.870) [29].

In this study, the concurrent validity of the K-BDRS was demonstrated based on its correlations with the HAMD, MADRS, and YMRS. The strong correlation coefficients of the K-BDRS with the other scales indicate that the K-BDRS accurately assesses depressive symptoms. Additionally, the mixed subscale score of the K-BDRS (Item 16–20) exhibited a strong correlation with the YMRS which indicates that the K-BDRS accurately assesses the mixed symptoms that frequently present in patients with bipolar depression. This is an important aspect of the K-BDRS because the rating of mixed features in bipolar depression is an area of great clinical significance.

The factor analysis of the K-BDRS revealed a three-factor solution that provided the best account of the present data. Although the factor loadings are different, the three-factor solution observed in the present study resembled the three-factor structure originally reported by previous studies [14]. As expected, the depressive subscales of the K-BDRS loaded onto psychological depressive symptoms (e.g., suicidal ideation, guilt, worthlessness, and helplessness) and somatic depressive symptoms (e.g., reduced activity and concentration and appetite disturbance). However, several items in the present study differed from those of the original study by Berk et al. [14]. In that study, anhedonia and flattened affect loaded onto psychological depressive symptoms but the present study found that anhedonia and flattened affect loaded onto somatic depressive symptoms. Because these symptoms are strongly related to reduced motivation and energy, it may be rational to assume that these features can be grouped under the somatic depressive symptoms factor.

Both of the depressive symptoms factors were strongly correlated with each other but weakly correlated with the mixed symptoms factor. All of the mixed subscales of the K-BDRS loaded together under the mixed symptoms factor, with the exception of lability. Lability is common in bipolar depression [10, 11] and similarly loaded onto both the psychological depressive and mixed symptoms factors. Psychotic symptoms are also included in the mixed symptoms factors, as demonstrated by Berk et al. [14]. This indicates that psychotic symptoms have a greater association with mixed features than with depressive symptoms. Our findings that the internal structure of the K-BDRS appears to be sensitive to mixed symptoms of bipolar depression are consistent with the previous reports [30].

The development of a Korean version of the K-BDRS is significant in several respects. The results of the present study confirmed the psychometric properties of the K-BDRS, which will enable both clinicians and researchers to use this instrument in the field to assess the severity and treatment responses of depressive symptoms in patients with bipolar disorder. Most importantly, the K-BDRS includes items that rate the mixed and atypical features of bipolar disorder, which may be overlooked by conventional depressive scales such as the MADRS and HAMD. Previous reports comparing unipolar and bipolar depression with the BDRS and the MADRS showed MADRS scores were unable to distinguish unipolar from bipolar depression. However, BDRS scores were significantly marked for the mixed subscale, suggesting that presence of mixed features during a depressive episode is in favour of bipolar depression [31].

The limitations of the present study were that the numbers of patients in the subgroups for the inter-rater and test-retest reliability ratings may have been insufficient. Additionally, the present study was cross-sectional and, therefore, it is difficult to determine whether the K-BDRS would be useful for the detection of changes in illness severity following treatment.

## Conclusions

The K-BDRS possesses good psychometric properties and is likely a valid and reliable tool for the assessment of depressive symptoms in patients with bipolar disorder. However, further studies are needed to evaluate the K-BDRS more fully. These studies should assess the applicability of the K-BDRS for various bipolar disorder subgroups as well as evaluate the scale’s utility for differentiating bipolar disorder from unipolar depression.

## Abbreviations

BDRS, the Bipolar Depression Rating Scale; CI, confidence interval; DSM-IV-TR, the Diagnostic and Statistical Manual of Mental Disorders, Fourth Edition, Text Revision; HAND, the 17-item Hamilton Depression Scale; ICC, intraclass correlation coefficients; K-BDRS, Korean version of the BDRS; MADRS, the Montgomery and Asberg Depression Scale; MINI, the Mini International Neuropsychiatric Interview; YMRS, the Young Mania Rating Scale
